# The role of magnetic resonance imaging in the management of brain metastases: diagnosis to prognosis

**DOI:** 10.1186/1470-7330-14-8

**Published:** 2014-04-22

**Authors:** Rasheed Zakaria, Kumar Das, Maneesh Bhojak, Mark Radon, Carol Walker, Michael D Jenkinson

**Affiliations:** 1 Department of Neurosurgery, The Walton Centre NHS Foundation Trust, Liverpool, UK; 2 Institute of Integrative Biology, University of Liverpool, Liverpool, UK; 3 Department of Neuroradiology, The Walton Centre NHS Foundation Trust, Liverpool, UK; 4 Institute of Translational Medicine, University of Liverpool, Liverpool, UK

**Keywords:** MRI, Brain metastasis, Perfusion, Diffusion, ADC, Spectroscopy, MRS, Biomarkers

## Abstract

This article reviews the different MRI techniques available for the diagnosis, treatment and monitoring of brain metastases with a focus on applying advanced MR techniques to practical clinical problems. Topics include conventional MRI sequences and contrast agents, functional MR imaging, diffusion weighted MR, MR spectroscopy and perfusion MR. The role of radiographic biomarkers is discussed as well as future directions such as molecular imaging and MR guided high frequency ultrasound.

## Introduction

Brain metastases are the most common central nervous system tumours in adults with a rising incidence due to the increased availability and utilisation of brain imaging and prolonged survival from primary cancers [1-3]. MRI is crucial in making the diagnosis, determining the best course of management, monitoring response to therapy and increasingly in trying to predict prognosis. Rather than reviewing each individual technique and its applications separately, as has been done elsewhere, the different clinical problems encountered in brain metastases are presented and the relevant MRI techniques which can be applied in each scenario addressed to give a practical summary [4-9].

## Review

### How many brain metastases are present?

**Figure 1:**
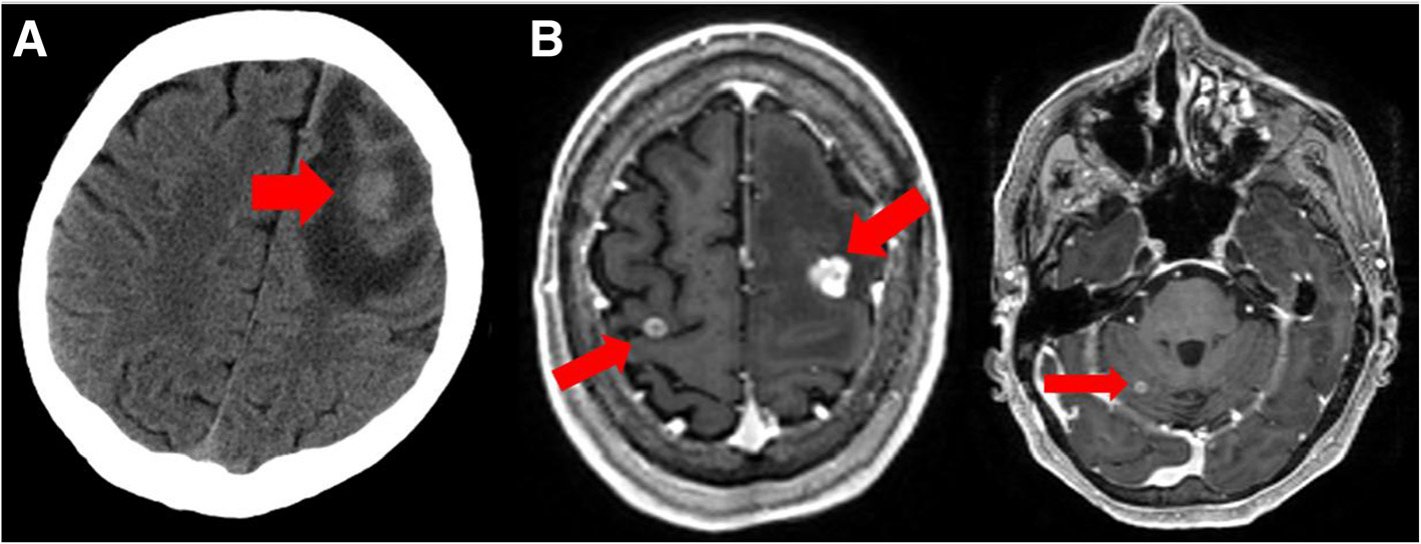
**A patient known to have melanoma presents with a first seizure and (A) unenhanced CT brain (32-slice scanner, 10 mm slices) taken in the emergency department shows an abnormality in the left frontal lobe with surrounding oedema – they were referred for possible neurosurgical intervention. (B)** T1 weighted MRI at 1.5 T with single dose (0.1 mmol/kg) gadolinium contrast detects this lesion but also delineates it further, allowing volume to be assessed accurately and in addition highlights two further areas of abnormality. After staging of the systemic disease and discussion with oncologists and surgeons, the patient was therefore treated with stereotactic radiosurgery to all three areas.

Accurately identifying the number, location and size of brain metastases is important to determine which interventions, if any, are appropriate for a patient. Multiple scoring systems used to predict prognosis take into account the number of lesions, for example the Recursive Partitioning Analysis or RPA classification [10-14]. With respect to detection, localisation and quantification, contrast enhanced MR has been widely demonstrated to be superior to both enhanced CT and non-enhanced MR [15-18] as illustrated in Figure 1. The recommended gadolinium dose in this context is 0.1 mmol/kg and whilst double or triple dose administration has been suggested to increase the detection of small lesions this causes increased false positives and a higher risk of nephrogenic systemic fibrosis therefore it remains an “off label” use [19-21].

*Could novel sequences and agents therefore increase detection without escalating the contrast dose?* Different gadolinium based agents, all of which may have slightly differing relaxivity profiles have been compared and these studies are summarised elsewhere. At present gadobutrol appears to identify the greatest number of lesions with the greatest contrast to noise ratio whilst having a lower risk of nephrogenic systemic fibrosis (along with the other “cyclic” structured gadolinium based agents [22]). MR sequences are developing rapidly but are not always explicitly evaluated against existing protocols. Magnetisation saturation transfer (MT) imaging has been directly compared to gradient echo T1 sequences and its addition reduced by half the standard dose of contrast needed for detection leading some to advocate it over ever increasing contrast doses [23,24]. More recently, 3D T1 weighted “spoiled gradient echo” (SPGR) and T2 weighted post contrast FLAIR sequences have been shown to detect submillimetric (<3 mm) abnormalities and more sensitively assess leptomeningeal disease [25,26]. The spatial resolution of the acquisition differs depending on the indication for the scan and this has important implications for detection and management which need to be kept in mind. For example if a treatment is decided upon with the multidisciplinary team and the patient but then a surgical or stereotactic radiosurgery (SRS) “planning” scan of higher spatial resolution is obtained, the latter will be more sensitive for detection of metastases than a conventional diagnostic scan and multiple, previously unseen lesions may be identified which render that treatment inappropriate [27].

### Is this solitary lesion a metastasis, an abscess or a high grade glioma?

A patient presenting with no known primary cancer and a solitary ring enhancing brain lesion may be suspected of having a brain metastasis, a primary cerebral tumour such as glioblastoma or a cerebral abscess, and despite careful history and exam, misdiagnoses may still occur [28]. Distinguishing abscess from tumour is perhaps the most clinically relevant question and in this regard diffusion-weighted MRI (DWI) has been most useful. Diffusion-weighted images detect free water movement and allow a surrogate of diffusion to be calculated for each voxel to generate apparent diffusion coefficient (ADC) maps. A large number of observational studies have shown that for a solitary cystic or necrotic contrast enhancing lesion, restriction of diffusion on pre-operative MRI is predictive of abscess [29-32]. Modifications to imaging protocols have increased sensitivity, including detection of early capsule formation during abscess development and even prediction of cellularity of abscesses at higher b-values and field strengths [33,34]. However, there are persistent cautions that some metastatic lesions may show restricted diffusion and mimic the appearances of abscesses, for example two different studies have reported this pattern for non-small cell lung carcinoma metastases, while others have also described it for lymphoma [35-38].

Distinguishing metastases from high grade glioma has proven more difficult using DWI although in theory the region of vasogenic oedema around metastases should show greater free diffusion than the more cellular, infiltrated region around a high grade glioma. Studies have varied in their cutoff for distinguishing the two pathologies as well as the methodology of how to take the reading (does one use the lowest ADC value, the mean of multiple measurements and which areas should one sample in the peri-tumoural region?) and there is no agreement about reliability [39-43]. Diffusion tensor imaging (DTI) is an enhancement of DWI with more diffusion gradients and directions used during acquisition. This allows more advanced metrics than just the average diffusion coefficient to be calculated and disruption of white matter tracts to be visualised. Again, the theory that high grade glioma is an invasive tumour which infiltrates white matter tracts whereas metastases deform them is *supported* but not consistently repeated by studies examining DTI metrics such as fractional anisotropy which may be superior to ADC alone [44-47].

**Figure 2:**
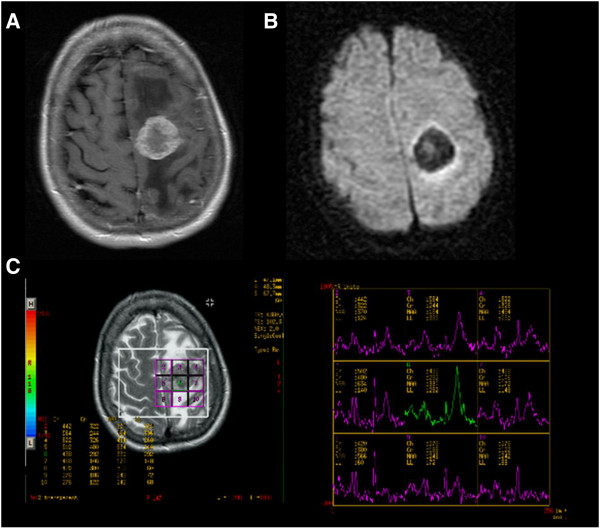
**An elderly patient was referred with hemiparesis and suspected to have a stroke.** MRI demonstrated a lesion in the left hemisphere which on **(A)** T1 weighted axial image post gadolinium at 1.5 T is shown to have a solid and ring enhancing portion. **(B)** The associated ADC map shows considerably reduced diffusion at the site of the solid portion of the lesion with increased diffusion due to vasogenic oedema in the white matter surrounding the mass. **(C)** Single voxel proton MRS of the lesion yields an abnormal spectrum with a large lipid and lactate peak, reduced NAA, reduced Cr and slightly elevated Cho. This pointed to a metastasis, glioma or lymphoma as opposed to an abscess. There was time to optimise the patient for surgery and begin steroid treatment before the lesion was resected and confirmed to be a renal cell carcinoma.

Further advanced techniques have therefore been combined with diffusion imaging in this context to try and increase sensitivity, most commonly MR spectroscopy [48]. Single and multivoxel magnetic resonance spectroscopy (MRS) provides information about the metabolic profile of specific regions in and around a lesion [49,50] as shown in Figure 2. Proton MRS has previously shown discriminatory power between high grade glioma and metastases by measurement of the choline/NAA ratio [51,52]. Novel spectroscopic markers continue to be investigated, including lipid and macromolecule concentrations by proton MRS [53] and phosphate metabolites by ^31^P MRS[54].

Further information on physiological activity can be gathered by combining with MR perfusion studies. MRI perfusion permits the generation of maps of relative cerebral blood flow (rCBF) and volume (rCBV) which are measures of vascularity. High grade glioma pathologically shows neovascularisation and infiltration of surrounding brain, hence the peritumoral rCBV is higher than for a metastasis [55,56]. Using the same dataset and different post processing techniques, diffusion susceptibility contrast or DSC MRI allows prediction of tissue permeability, a measure of blood-brain barrier disruption and this accordingly increases around a metastasis, where there is increased capillary permeability and therefore vasogenic oedema [57,58].

In summary, multimodal MR used singly or in combination has improved our ability to distinguish metastases from primary cerebral tumours or abscesses. Despite the application of these advanced MR techniques of diffusion, perfusion and spectroscopy to solve this clinical problem [59] it remains a fact that for accessible, larger lesions surgery is often undertaken and tissue diagnosis obtained *regardless* in order to move forward treatment. The role and evidence for MRI in surgical planning is therefore the next area to consider.

### What information does the neurosurgeon need and how can MRI provide it?

**Figure 3:**
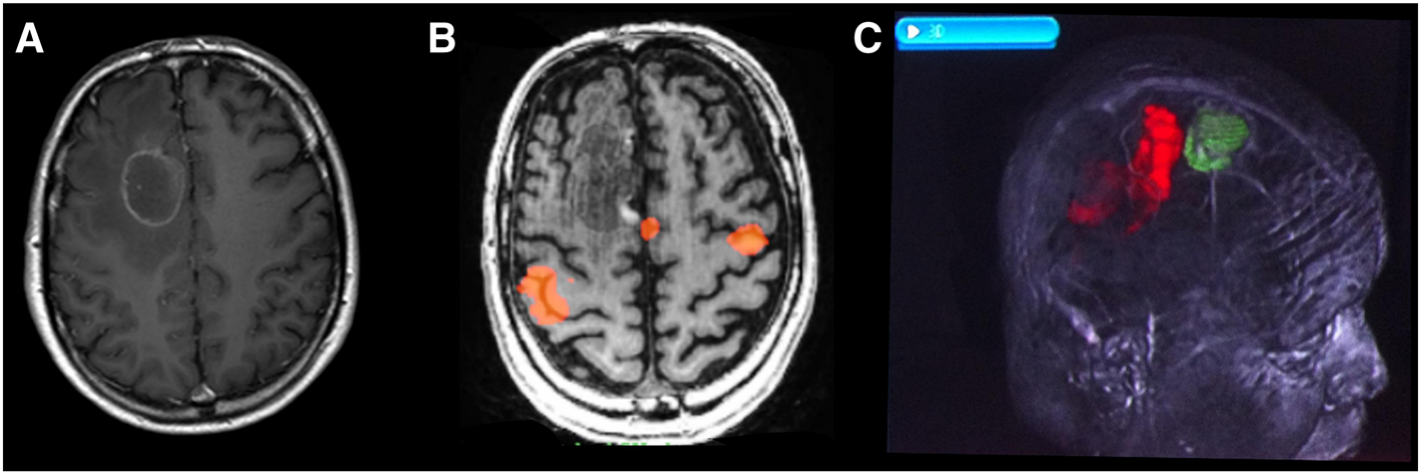
**A patient known to have breast cancer with a manually dexterous job presented with intermittent left hand and arm weakness and was found (A) to have a solitary ring enhancing lesion in the premotor area on T1W MRI with gadolinium. (B)** functional MRI performing a hand tapping and gripping task determined the location of hand function and this was used as the “seed” region of interest on a DTI scan to produce a representation of the motor tracts. **(C)** these were used to generate a 3D object and fused with an anatomical planning scan (1 mm slices) using commercially available software (StealthViz™ with StealthDTI™ by Medtronic, running on an S7 workstation) to produce images that were used intraoperatively for image guided resection, avoiding the tracts (shown in red, with tumour rendered in green).

Image guided surgery allows the surgeon to plan the safest route to the tumour preoperatively and to maximise safe resection intraoperatively. Conventional post contrast T1 weighted images with thin slice protocols are generally acquired for image guidance software registration and the increased spatial resolution of such sequences compared to the usual diagnostic scans (and thus potential to detect more lesions than first seen) has been highlighted in the discussion of detection. Diffusion imaging allows images of white matter tracts and their relationship to tumour to be delineated. DTI acquisitions, in addition to generating quantitative parameters, can permit tractography by estimating the directionality of fibre tracts, permitting the tracking of fibre bundles in 3D space. The methodology for this post processing varies and a number of non-proprietary software solutions in addition to those supplied by manufacturers are available. Further refinements to the methodology, including use of higher order algorithms to resolve ambiguous directionality on voxels may further increase reliability [60]. Functional MR detects changes in blood oxygen level or BOLD signal in metabolically more active areas during application of a stimulus or performance of a task. This is particularly useful to localise language areas or motor cortex. Few series have examined the role of fMRI in metastasis resection alone but in those that have significant benefits in terms of motor recovery, preservation and therefore quality of life have been demonstrated [61]. Commercially available software can integrate functional and tractography sequences into a single merged dataset for use in theatre as shown in Figure 3, with improved outcomes for series looking exclusively at metastases [62,63].

### Where is the original, primary cancer?

In cases of multiple metastases or solitary lesions with no known primary, MRI may give useful diagnostic clues as to the original tumour. Some primaries may have particular signal characteristics even on conventional MR. For example melanoma metastases may show high signal on non-enhanced T1 sequences due to the effect of melanin and mucinous metastases may show low T2 signal compared with the expected hyper-intensity on this sequence. The metabolic profile has been investigated for metastases of differing primary tumours as well as the surrounding brain with no abnormal spectra reported outside of the lesion itself. Metabolic features on MRS have shown limited value in predicting primary type. However, raised mobile lipid content has been proposed as a weak sign of a colonic origin for metastasis [64].Others have tried to use the diffusion characteristics of particular metastases to distinguish the primary and whilst it has been shown that ADC values are higher in well differentiated adenocarcinoma metastases than poorly differentiated types this may simply reflect cellularity and could not predict the primary, only suggest a narrower differential [36]. Likewise in a series with a variety of primary cancers including lung and breast, restricted diffusion could not be correlated with any primary nor could ADC predict primary pathology [35]. Spectroscopy was combined with perfusion MR to show that differences in choline-creatine ratios between metastases of lung and breast cancer correlated with differences in relative cerebral blood volume but tissue was not available for comparison to look for a unifying pathological basis [65].

In summary, MRS may be used to distinguish primary tumour origin. However more than one advanced MR metric may need to be combined in order to robustly generate models that differentiate the primary lesion and though these studies would likely be retrospective, ideally some image guided correlation of regions of interest on the advanced MR with the final tissue samples is needed, as has been performed for glioma [45].

### Are these brain metastases responding to chemo/radio-therapy?

**Figure 4:**
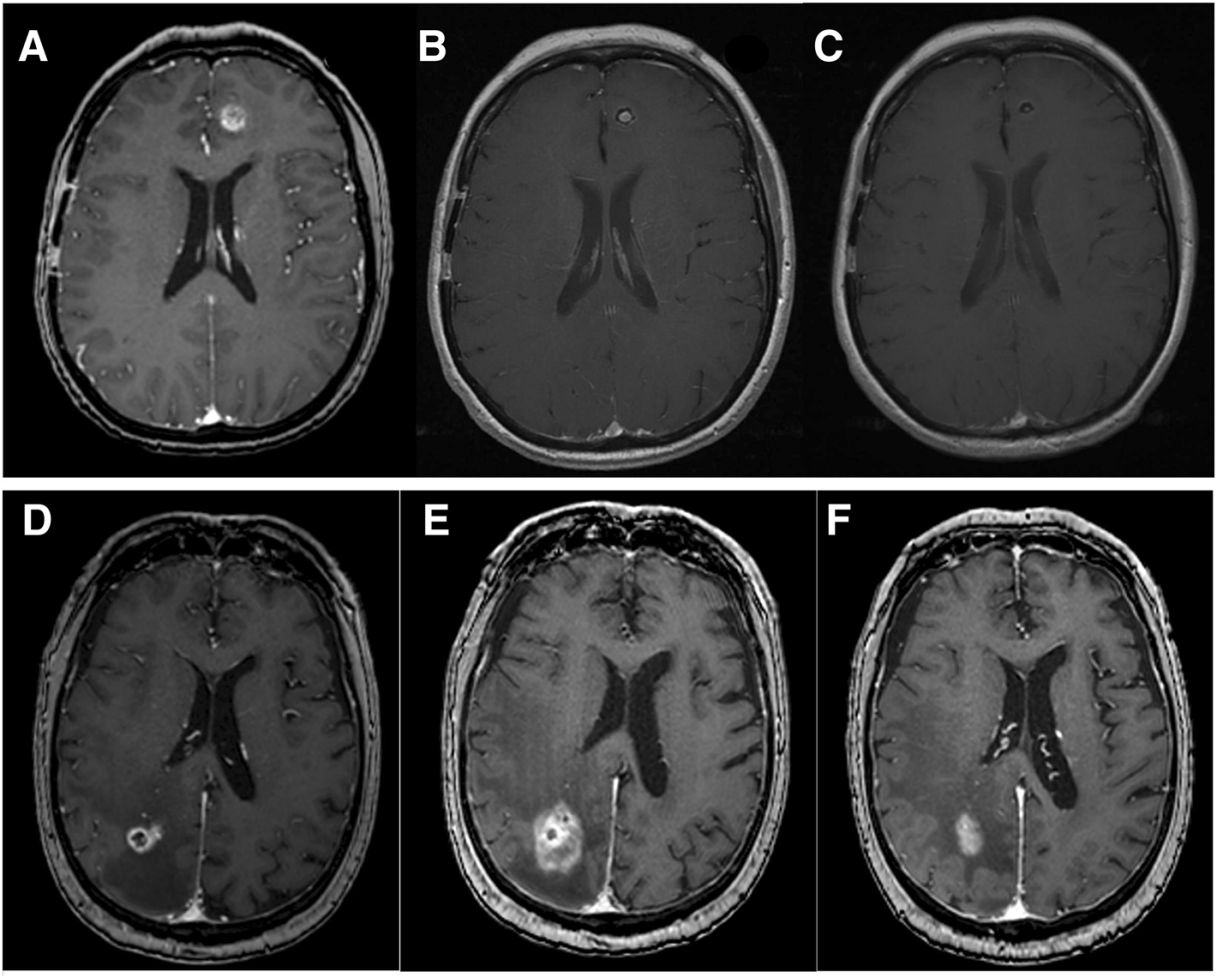
**Monitoring of treatment response after stereotactic radiosurgery.** A patient with known metastatic melanoma underwent treatment to a left frontal lesion **(A)** and at 3 months **(B)** the characteristic changes of a blurred margin and necrosis followed by formation of a glial scar at 6 months **(C)** are seen. However in a similar appearing patient **(D)** with a right parietal lesion from metastatic renal cell carcinoma the lesion shows more florid changes at 3 months **(E)** with increased contrast enhancement at the margins and the possibility of progression as opposed to radionecrosis is raised. Multi-modal techniques described in the text may show promise in deciding how to proceed at this stage. The patient was well and on monoclonal antibody chemotherapy, with observation the lesion eventually regressed at 6 months **(F)** and is currently stable.

Beyond diagnosis, MRI may be used to monitor response to treatment as part of clinical and radiological follow up. This may be immediate, as in post-operative MRI to determine if there has been a complete resection or delayed, i.e. has the metastasis responded chemo- or radio-therapy. In general, conventional sequences are utilized but one area of particular clinical interest in following cases up is ongoing or increasing enhancement following surgery or stereotactic radiosurgery to a metastasis and whether this represents radio-necrosis or recurrence [66]. Standard patterns of changes in brain metastases are seen on conventional MRI with perilesional oedema, central hypointensity on T2 weighted imaging at 2-6 months followed by blurring of the enhancement margin, reduction in enhancing volume over time and formation of a glial scar [67-69]. These changes and difficulty in interpreting responses are illustrated in Figure 4. Although attempts have been made to build discriminatory measures from conventional MRI [70] performance has proven disappointing [71]. Blood flow would be expected to differ significantly between areas of necrosis and recurrence. MR perfusion has shown higher rCBV in recurrent metastases after radiosurgery compared to areas of radionecrosis and reduction in CBF over time for treated lesions [72,73]. Furthermore, the first reading of CBF following treatment was highly predictive of the final response, even though pre-operative readings could not predict this. Diffusion imaging may reflect cellularity and ADC readings from metastases treated with radiosurgery, taken immediately post treatment may be tracked to determine if the lesion is responding to therapy, manifest as increasing ADC as compared to a recurring or necrotic lesion where the changes in ADC differ. The initial ADC value may also predict final response to the treatment [74]. In conjunction with perfusion MRI, this suggests the novel application of advanced MR readings, not just as diagnostic tools but as predictors of future treatment response or biological markers.

### Could radiographic biomarkers predict survival?

As described in many studies here, various metrics or measurements may be taken from the different advanced MR images described using the device workstation or non-proprietary software. A biological marker or “biomarker” is strictly any such reading which is used for diagnostic or prognostic purposes. Even conventional MR may provide such markers and it has been shown that for solitary metastases in one series, the degree of oedema on the preoperative T2 weighted scan was related to the degree of angiogenesis, brain invasiveness and overall survival with reduced oedema surprisingly being a worse prognostic factor [75]. The role of ADC and CBF in predicting response of metastases to SRS has been discussed in the preceding section. In a recent study preoperative DWI and ADC readings were assessed in single metastases which were subsequently resected. A reduced overall survival was found irrespective of adjuvant treatments and there was a denser reticulin (stromal) matrix in patients with low tumour ADC compared to the group average [76]. These measures could potentially be incorporated into the prognostic models mentioned such as the recursive partitioning score (RPA) or graded prognosis assessment (GPA) score; widely validated predictors of survival in brain metastases patients which combine clinical information such as age, status of the primary cancer and extracranial disease amongst others [10,77]. Further standardisation of the post processing and measurement workflow is needed, however, before MRI metrics could be confidently used in clinical practice.

### What are the emerging directions of MRI in brain metastases management?

As cancer diagnostics and therapeutics become increasingly related three areas of emerging MR technology with practical applications to brain metastases stand out. Integration of cancer staging via PET-MR offers one potential means of incorporating further functional data in real time without losing all the knowledge already acquired about the behaviour and characteristics of metastases on MRI [78]. Novel contrast agents including those that can identify molecular targets are in development in animals and an agent based on iron oxide particles which binds to a vascular cell adhesion molecule common to human metastases has been used to visualise micro-metastases at MRI, suggesting the possibility of diagnosing and possibly treating brain metastases long before they were previously even detected [79]. Finally the application of MR as an intraoperative tool for guiding minimally invasive therapies such as laser coagulation or high intensity focused ultrasound is no longer conceptual, with several small series of treated tumours including metastases [80,81]. MR technology will continue to enhance diagnosis but is now being used to predict prognosis and being incorporated into the treatment of metastases too [82].

## Conclusions

This article has summarised the current evidence for the *practical* application of advanced MRI techniques including diffusion weighted imaging, tractography, perfusion studies, functional MRI and MR spectroscopy in common clinical scenarios relating to brain metastases. Previously the literature has predominantly focused on applying these modalities to intrinsic brain tumours such as glioma but there appears to be an increasing recognition of the burden of metastatic brain disease and the need for novel applications of MR technology to solve the sort of practical clinical problems described and provide more prognostic information. It may be that this occurs by development of further conventional sequences and better contrast media, including at a molecular level, or by combination of the existing possibilities to generate multimodal datasets. Evidence of practical, clinical utility specific to metastases needs to be gathered at each stage to justify the development of - and determine the best use of - these expensive resources.

## Abbreviations

MRI: Magnetic resonance imaging; ADC: Apparent diffusion coefficient; ROI: Region of interest; MRS: Magnetic resonance spectroscopy; DWI: Diffusion weighted imaging; FLAIR: Fluid attenuated inversion recovery; rCBV: Relative cerebral blood volume; CBF: Cerebral blood flow; fMRI: Functional magnetic resonance imaging; RPA: Recursive partitioning analysis; GPA: Graded prognostic assessment; SRS: Stereotactic radiosurgery; DTI: Diffusion tensor imaging; DSC: Dynamic susceptibility contrast.

## Competing interests

The authors declare that they have no competing interests.

## Authors' contributions

RZ drafted the article with assistance from KD, MB and MR. MR and MB assisted with finding figures. CW, MDJ, MR significantly edited the article. All authors read and approved the final manuscript.
